# Role and Relevance of Warm Saline Mouth Rinses in Contemporary Dental Practice: A Narrative Review

**DOI:** 10.7759/cureus.113591

**Published:** 2026-07-29

**Authors:** G Harshini, B Roshan Arbaaz, Sowjanya Bandlamudi, Subramanian Adalarasan, S Peter, S Kangaiyan, Thangadurai Maheswaran

**Affiliations:** 1 Oral and Maxillofacial Pathology, Sri Ramakrishna Dental College and Hospital, Coimbatore, IND; 2 Periodontics, Sri Venkateshwaraa Dental College, Puducherry, IND; 3 Anatomy, Shri Sathya Sai Medical College and Research Institute, Sri Balaji Vidyapeeth (Deemed to be University), Puducherry, IND; 4 Oral and Maxillofacial Surgery, Government Dental College and Hospital, Chidambaram, IND; 5 Prosthodontics, Sri Venkateshwaraa Dental College, Puducherry, IND; 6 Prosthodontics, Adhiparasakthi Dental College and Hospital, Melmaruvathur, IND; 7 Oral and Maxillofacial Pathology, Adhiparasakthi Dental College and Hospital, Melmaruvathur, IND

**Keywords:** alveolar osteitis, chlorhexidine, oral hygiene, oral mucositis, periodontal therapy, saline mouthwash, wound healing

## Abstract

Warm saline mouth rinsing is one of the oldest and most widely practiced adjuncts in dentistry; however, its evidence base has been substantiated only recently, both mechanistically and clinically. In vitro studies have shown that short-term rinsing with hypertonic sodium chloride promotes gingival fibroblast migration and extracellular matrix protein expression via a chloride-dependent pathway, offering a cellular explanation for its long-observed wound-healing benefits. Clinically, saline rinsing has consistently been shown to be effective in reducing alveolar osteitis following tooth extraction, with benefits comparable to those of several antimicrobial rinses, and randomized evidence supports its use as a practical, postoperative default. In oncology, saline is part of standard basic oral care during radiotherapy and chemotherapy, although systematic reviews have found evidence for its independent efficacy against oral mucositis to be inadequate or conflicting, with several herbal and iodine-based comparators showing comparable or superior symptomatic benefits. In critical care, saline-based oral hygiene protocols show a favorable mortality and microbial diversity profile relative to chlorhexidine in mechanically ventilated patients, despite chlorhexidine’s superior effect on ventilator-associated pneumonia incidence. Comparative trials across periodontal and antimicrobial contexts generally position saline below chlorhexidine and several herbal formulations for plaque and gingival index outcomes, although its safety profile makes it a suitable substitute for patients intolerant to chlorhexidine. Limitations include a thin mechanistic evidence base, potential effects on enamel surface hardness with prolonged use, and reliance on small and heterogeneous studies. For dental practitioners, warm saline mouth rinsing remains a safe, low-cost, and evidence-based adjunct across surgical, periodontal, oncological, and critical care contexts and is best positioned as a first-line or complementary rinse rather than a substitute for targeted antimicrobial therapy, where high-potency plaque or pathogen control is required.

## Introduction and background

Warm saline mouth rinsing is among the oldest and most widely recommended adjuncts in dental practice, with documented use in oral hygiene tracing back thousands of years [1]. Despite its long history, rigorous mechanistic evidence of its benefits has only recently been reported. In vitro studies have demonstrated that short-term rinsing with 0.9-1.8% sodium chloride promotes human gingival fibroblast migration and upregulates extracellular matrix proteins central to wound repair [1]. Clinically, saline rinsing is most robustly supported in the context of postoperative extraction care, where randomized trials have shown that it significantly reduces the incidence of alveolar osteitis relative to no rinsing, with an efficacy comparable to that of several antimicrobial alternatives [2,3]. Its role extends well beyond routine extractions, spanning periodontal wound care, supportive oncology practice during radiotherapy and chemotherapy-induced oral mucositis, and oral hygiene protocols in mechanically ventilated intensive care patients, where a Cochrane review and a subsequent network meta-analysis both identified measurable, albeit modality-specific, benefits [4,5]. Warm water is traditionally recommended in clinical practice because it improves patient comfort and facilitates dissolution of salt; however, the available biological and clinical evidence primarily relates to the effects of saline itself rather than the temperature of the solution.

Nonetheless, the efficacy of saline relative to chlorhexidine and other antimicrobials or herbal rinses is inconsistent across different indications. Systematic reviews evaluating basic oral care for chemotherapy-induced mucositis in both adult and pediatric populations have found the evidence for saline to be inadequate or conflicting [6,7], while comparative periodontal trials have generally shown that saline underperforms compared to chlorhexidine and several herbal formulations on plaque and gingival indices [8,9]. Simultaneously, concerns regarding the disruption of oral microbial diversity by chlorhexidine and its non-selective antimicrobial action have renewed clinical interest in saline as a lower-risk alternative, particularly in patients who are intolerant to chlorhexidine and are critically ill [10,11]. A comprehensive synthesis addressing the mechanistic rationale of saline and its comparative clinical performance across these varied contexts, oral surgery, oncology, critical care, and general periodontal practice, has not been consolidated in a single narrative review. Therefore, this review aims to examine the physiological basis for warm saline mouth rinsing, its evidence-based applications across these clinical domains, its performance relative to antimicrobial and herbal comparators, and the limitations that should inform its continued use in contemporary dental practice.

## Review

Methodology

This narrative review was undertaken to summarize the current evidence on the role of warm saline mouth rinses in contemporary dental practice. A targeted literature search was conducted in PubMed in June 2026 using combinations of the following terms: warm saline mouth rinse, salt water mouthwash, oral hygiene, wound healing, alveolar osteitis, oral mucositis, chlorhexidine, periodontal therapy, and ventilator-associated pneumonia (VAP). Only English-language publications were included in the review. A total of 22 references were included in this narrative review. Studies were purposively selected based on their relevance to the review objectives, with emphasis on recent evidence while incorporating landmark studies where appropriate. The evidence included systematic reviews, meta-analyses, randomized and other clinical studies, consensus statements, and clinical practice guidelines. Owing to the narrative nature of this review, no predefined systematic inclusion or exclusion criteria, protocol registration, or formal risk-of-bias assessment were applied.

Physiological basis and mechanism of action

The biological rationale for saline mouth rinsing is based on a single mechanistic study that directly examined the cellular effects on oral tissues. Using primary human gingival fibroblasts, this in vitro study found that short-term rinsing with 0.9% to 1.8% sodium chloride significantly promoted fibroblast migration without affecting proliferation and that this concentration range also upregulated type-I collagen and fibronectin expression, both of which are integral to extracellular matrix remodelling during wound healing [1]. Mechanistically, the same study identified chloride ions, rather than sodium ions, as the essential mediator of this migratory effect, as rinsing with potassium chloride reproduced the stimulatory action, whereas sodium and potassium phosphate salts did not. This further demonstrated the upregulation of focal adhesion kinase and reorganization of F-actin cytoskeletal filaments, consistent with enhanced cell motility [1]. Notably, human oral keratinocytes did not respond to the same saline concentrations, indicating that the wound-healing benefit of saline rinsing may be cell type-specific, preferentially acting on connective tissue fibroblasts rather than epithelial cells [1]. The same study cautioned that excessively high NaCl concentrations, specifically 7.2%, reversed this beneficial effect and impaired migration, underscoring that the commonly recommended concentration of approximately one teaspoon of salt per cup of water approximates the physiologically optimal hypertonic range rather than an arbitrary folk convention [1]. This mechanistic study, although limited in scope, provides one of the strongest available cellular explanations for the long-observed clinical benefit of saline rinsing in gingival and mucosal wound contexts, although the findings await confirmation in additional cell models and in vivo systems.

Oral surgery, extraction, and periodontal wound care

Warm saline mouth rinses are commonly used in postoperative care following dentoalveolar surgery, primarily because of their consistent association with reduced alveolar osteitis rates. A systematic review and meta-analysis of eight randomized trials found that warm saline mouth rinses showed no statistically significant difference in the incidence of alveolar osteitis compared with antimicrobial rinses, while also reducing complications relative to no rinsing; however, the authors cautioned that most of the included trials had a high risk of bias [2]. This near-equivalence to antimicrobial agents is clinically important because it supports saline as a low-cost, low-toxicity alternative that does not compromise protective efficacy. A randomized trial in patients undergoing non-surgical tooth extraction reinforced this signal directly: instructing patients to gargle with warm saline reduced the incidence of alveolar osteitis to 2.5% compared to 25% in patients given no such instruction, and a twice-daily regimen performed as well as a six-times-daily regimen while being more convenient for patients [3]. Compliance is a decisive factor in mediating these benefits. In a prospective study of patients following intra-alveolar molar extraction, compliance with a warm saline rinse regimen was associated with a markedly lower ratio of alveolar osteitis cases relative to non-compliant patients, and warm saline rinse alone was the most effective single regimen for preventing localized alveolar osteitis among the studied regimens [12]. Mechanistically, one report proposed that irrigation of the extraction socket with saline in increasing volumes progressively lowers dry socket incidence, likely by clearing debris and food particles that would otherwise disrupt the formation of fibrin clots [12].

Beyond extraction sockets, saline has also served as a reference comparator against which newer topical wound-healing agents are benchmarked, a role that has generated useful data on their relative efficacies. In a randomized controlled trial comparing myrrh mouthwash with saline after tooth extraction, the myrrh group showed significantly greater improvement in postoperative surgical site edema, tenderness, and socket size, although no significant between-group differences in pain were detected [13]. The trial’s discussion noted that myrrh’s anti-inflammatory action, mediated through inhibition of nitric oxide, prostaglandin E2, and tumor necrosis factor-alpha, may explain the accelerated resolution of local inflammatory signs relative to saline alone [13]. Similarly, a trial evaluating the efficacy of sucralfate oral rinse against normal saline in secondary-healing intraoral surgical wounds found that sucralfate produced significantly greater reductions in pain scores on postoperative days three and seven, although wound grade and maximal wound length reduction did not differ significantly between groups, indicating that saline’s analgesic profile, while adequate, can be outperformed by agents with a specific mucosal coating action [14]. An older animal study comparing chlorhexidine, a phenolic mouthrinse, an amine/stannous fluoride rinse, and saline on excisional palatal wounds found that epithelialization proceeded at a comparable or superior rate with chlorhexidine gel and the phenolic rinse relative to saline, although none of the agents tested, including saline, impaired wound closure [15]. Collectively, these comparative data indicate that saline is neither uniquely superior nor inferior for wound closure kinetics; however, its low cost, absence of cytotoxicity, and ease of preparation sustain its role as a practical first-line postoperative rinse, particularly in cases where compliance and accessibility outweigh marginal gains from specialized agents.

Applications in oncology: oral mucositis management

Saline-based rinses have long been part of “basic oral care” protocols recommended for patients undergoing radiotherapy or chemotherapy for cancer, although the strength of the supporting evidence varies considerably by outcome and population. A systematic review conducted by the Multinational Association of Supportive Care in Cancer (MASCC) and the International Society of Oral Oncology (ISOO) evaluated seven interventions, including normal saline, and concluded that while oral care protocols could be recommended across treatment modalities, the evidence for normal saline was inadequate or conflicting, precluding a definitive guideline in either direction [6]. A parallel systematic review focused on pediatric patients undergoing chemotherapy reached a similarly cautious conclusion, reporting that among the rinses examined, including chlorhexidine, sodium bicarbonate, and saline, none had been shown to be definitively effective in preventing chemotherapy-induced oral mucositis [7]. These findings temper any assumption that saline confers a robust prophylactic benefit against mucositis, distinguishing its established role in wound healing from its equivocal role in oncology-specific mucosal protection.

Direct comparative trials further illustrate this ambiguity. A double-blind randomized trial comparing an in-house iodine solution against normal saline solution in head and neck cancer patients undergoing concurrent chemoradiation found no statistically significant difference in the weekly Oral Mucositis Assessment Scale score, pain score, or impact on swallowing, and the onset and duration of mucositis were also comparable between groups, although the trial was underpowered given its small sample size [16]. A separate two-part trial compared a herbal mouthwash containing five plant extracts with a soda saline mouthwash in patients undergoing radiotherapy for head and neck cancer. The herbal formulation produced a significantly greater reduction in the severity of radiation-induced mucositis and a lower requirement for analgesics, whereas pain intensity, xerostomia, and oral hygiene were similar between groups [17]. This suggests that saline alone, although widely used as a standard-of-care comparator, may be outperformed by adjunctive anti-inflammatory formulations on certain severity endpoints, even when baseline symptomatic relief is comparable to that of saline alone.

Emerging microbiome-focused research has begun to mechanistically contextualize these results. A randomized trial in pediatric oncology patients comparing a supersaturated calcium phosphate rinse (Caphosol) with saline during chemotherapy found that although no life-threatening mucositis occurred in either group, chemotherapy induced measurable shifts in oral bacterial composition, and mucositis was associated with distinct microbial profiles, including elevated *Stenotrophomonas* and *Leptotrichia* species, independent of the rinse used [18]. This indicates that the driver of mucositis severity may be as much a function of treatment-induced dysbiosis as the antiseptic properties of any single rinse, a nuance that helps explain the inconsistent superiority of saline over comparator agents in the clinical trials. Taken together, the oncology literature supports the continued inclusion of saline in basic oral care regimens on the grounds of safety, tolerability, and low cost, but does not support claims of independent prophylactic efficacy against oral mucositis.

Applications in critical and hospital care settings

In intensive care, oral hygiene protocols are primarily directed at reducing the risk of VAP, and saline occupies a specific niche within the broader landscape of antiseptic options. A major Cochrane systematic review of oral hygiene care in critically ill, mechanically ventilated patients found high-quality evidence that chlorhexidine mouthrinse or gel reduced the risk of VAP from approximately 25% to 19%, but also reported weaker evidence, drawn from four studies, that saline rinse was more effective than saline swab in reducing VAP, alongside separate weak evidence favoring povidone-iodine mouthrinse over saline or placebo [4]. Notably, the same review found no significant difference between chlorhexidine and placebo or usual care for mortality, duration of mechanical ventilation, or length of intensive care unit (ICU) stay, underscoring that reductions in VAP incidence do not automatically translate into improved hard outcomes [4]. A more recent network meta-analysis of 14 randomized trials in ICU patients extended this picture, finding that saline administration was associated with a significant reduction in ICU mortality compared with no mouthwash use, whereas antimicrobial agents such as chlorhexidine showed no mortality benefit, and oxidizing agents showed only a non-significant trend toward reduced VAP incidence [5]. This observed association with reduced mortality for saline, while requiring confirmation in larger high-quality trials, distinguishes it from antiseptic rinses, the benefits of which appear to be confined to microbiological or infection incidence endpoints rather than survival rates.

Concerns regarding the safety profile of chlorhexidine in this setting have further strengthened the rationale for using saline as an alternative antiseptic. A single-center randomized trial in elderly mechanically ventilated ICU patients comparing chlorhexidine with saline oral care found that chlorhexidine produced a significant decrease in oral bacterial diversity after one week; however, this did not translate into any significant difference in VAP incidence, duration of ventilation, ICU stay, or 28-day mortality relative to saline, leading the authors to caution that chlorhexidine’s disruption of microbial balance warrants caution despite its antiseptic potency [10]. Taken together, the critical care literature does not position saline as a uniformly superior agent but as a low-risk option whose mortality and diversity-preserving profile compares favorably with the disruptive, non-selective antimicrobial action of chlorhexidine, particularly in vulnerable, immunologically compromised populations, where preserving a balanced oral microbiota may be systemically relevant.

Comparative efficacy versus antimicrobial and herbal rinses

When benchmarked directly against chlorhexidine, the antimicrobial gold standard in dentistry, saline generally shows a smaller antiplaque and anti-inflammatory effect, although the clinical gap narrows in specific populations and for specific outcomes. In a randomized trial comparing a probiotic mouthwash, 0.2% chlorhexidine, and saline in patients with chronic gingivitis, both probiotic and chlorhexidine rinses produced significantly greater reductions in plaque index, gingival index, and oral hygiene index than saline, with no significant difference between the probiotic and chlorhexidine groups [8]. A crossover trial evaluating alcohol-free and alcohol-based essential oil mouth rinses against chlorhexidine and a saline placebo in a three-day plaque regrowth model similarly found that both essential oil formulations outperformed saline in inhibiting plaque regrowth, although chlorhexidine remained superior to all other agents tested [9]. These findings are consistent with the general positioning of saline below active antimicrobial agents for plaque control, although exceptions exist.

Notably, the relative standing of saline improves in patients who cannot tolerate chlorhexidine or require anti-inflammatory rather than antiplaque benefits. A randomized trial in young adults with self-reported chlorhexidine allergy found that 2% saline rinses and a herbal mouthwash produced comparable postoperative reductions in plaque index, gingival index, and probing depth after non-surgical periodontal therapy, positioning saline as a suitable substitute prescription in this subgroup [11]. A four-day non-brushing biofilm regrowth trial comparing electrolyzed saline, chlorhexidine, and placebo found that while the biofilm-inhibitory effect of chlorhexidine remained superior, electrolyzed saline significantly reduced biofilm accumulation compared with placebo and, importantly, preserved commensal bacterial populations that were suppressed by the broad-spectrum action of chlorhexidine [19]. This selective dysbiosis-sparing profile suggests that specially formulated saline preparations may offer a favorable ecological trade-off, even when the raw antiplaque potency lags behind that of chlorhexidine. In an unrelated but clinically relevant context, a randomized trial assessing mouth rinses for reducing salivary SARS-CoV-2 viral load found that povidone-iodine mouth rinse produced a significant short-term reduction in viral load compared with saline, whereas chlorhexidine forms showed inconsistent effects, reinforcing that antiseptic potency against specific pathogens does not generalize uniformly across all antimicrobial agents [20]. Table 1 summarizes the reported comparative outcomes of saline against key antimicrobial and herbal rinse comparators in these trials.

**Table 1:** Comparative efficacy of saline versus antimicrobial and herbal mouth rinses across clinical trial contexts. CHX = chlorhexidine; RCT = randomized controlled trial; PI = plaque index; GI = gingival index; OHI-S = oral hygiene index-simplified; NSPT = non-surgical periodontal therapy; EOS = electrolyzed saline; PVP-I = povidone-iodine. Source: Original table created by the authors based on data synthesized from references [8,9,11,15,19,20].

Comparator	Population/Model	Key outcome vs. saline	Reference
CHX gel/solution	Excisional palatal wounds (animal)	CHX gel showed superior epithelialization rate; saline did not impair healing	[15]
CHX 0.2%	Chronic gingivitis (RCT)	CHX and probiotic both significantly reduced PI/GI/OHI-S vs. saline	[8]
Herbal mouthwash (2% saline arm)	Post-NSPT periodontal inflammation, CHX-allergic patients	Saline and herbal rinse comparably effective; suitable CHX alternative	[11]
Electrolyzed saline (EOS) vs. CHX	4-day non-brushing biofilm regrowth	CHX superior for biofilm inhibition; EOS preserved commensal bacteria	[19]
Essential oil mouthrinses vs. CHX	3-day plaque regrowth (crossover)	CHX most effective; essential oils outperformed saline placebo	[9]
PVI-I vs. CHX forms	Salivary SARS-CoV-2 viral load	PVP-I significantly reduced viral load; CHX forms inconsistent	[20]

Limitations and future directions

Despite its favorable safety profile, warm saline rinsing has certain limitations that require clinical attention in future studies. An in vitro study assessing the effects of five common mouthwashes, including warm saline, on extracted permanent teeth found that saline produced a significant decrease in enamel surface hardness after seven days and the greatest increase in surface roughness among all agents tested, suggesting that even ostensibly innocuous saline rinses are not without potential effects on hard dental tissue with prolonged or repeated use [21]. This finding tempers the assumption that saline is biologically inert and indicates the need for further investigation into the optimal rinsing frequency and duration, particularly for patients using saline rinses as a long-term adjunct rather than during a short, postoperative course. A broader literature review of treatments for recurrent aphthous stomatitis found that non-pharmacological therapies, including low-level laser therapy and various natural compounds such as aloe vera and curcumin, generally offered a favorable risk-benefit ratio and, in several trials, outperformed standard supportive rinses on pain and healing-time endpoints, indicating that saline’s role in ulcerative oral disease may increasingly be complemented rather than replaced by these adjunctive modalities [22]. Across the specialty areas reviewed, the evidence base for saline remains constrained by small trial sizes, heterogeneous concentrations and rinsing protocols, and a predominance of surrogate rather than patient-centered outcomes. Future well-powered trials with standardized saline concentrations, rinsing frequency, and duration, alongside mechanistic studies extending beyond the single available fibroblast model, are needed to place the role of saline in contemporary dental practice on a firmer evidentiary footing. Furthermore, as this was a narrative review, selective inclusion of the literature may have introduced literature selection bias.

## Conclusions

Warm saline mouth rinsing remains a durable, low-cost fixture of contemporary dental and hospital practice, supported by a plausible cellular mechanism, consistent benefit in reducing alveolar osteitis after tooth extraction, and a favorable safety and microbial preservation profile relative to chlorhexidine in vulnerable populations such as mechanically ventilated ICU patients. Its role in oral mucositis prevention during cancer therapy is less well established, with systematic reviews finding insufficient evidence to recommend it over alternative or adjunctive agents. However, its antiplaque potency consistently lags behind that of chlorhexidine and other herbal formulations. For clinicians, the practical takeaway is that saline should continue to be recommended as a safe, first-line postoperative and adjunctive rinse, particularly where cost, tolerability, or chlorhexidine intolerance are limiting factors, while active antimicrobial or anti-inflammatory agents should be reserved for indications such as plaque control in periodontitis or high-risk mucositis prevention, where the evidence for saline alone is insufficient.

## References

[1] Huynh NC, Everts V, Leethanakul C, Pavasant P, Ampornaramveth RS (2016). Rinsing with saline promotes human gingival fibroblast wound healing in vitro. PLoS One.

[2] Adekunle AA, Egbunah UP, Erinoso OA, Adeyemo WL (2021). Effectiveness of warm saline mouth bath in preventing alveolar osteitis: a systematic review and meta-analysis. J Craniomaxillofac Surg.

[3] Stewart M, Levey E, Nayyer N (2015). Salt water mouthwash post extraction reduced post operative complications. Evid Based Dent.

[4] Hua F, Xie H, Worthington HV, Furness S, Zhang Q, Li C (2016). Oral hygiene care for critically ill patients to prevent ventilator-associated pneumonia. Cochrane Database Syst Rev.

[5] He Q, Peng Z, He C, Zhang C, Hu R (2025). Effect of different mouthwashes on ventilator-related outcomes and mortality in intensive care unit patients: a network meta-analysis. Aust Crit Care.

[6] McGuire DB, Fulton JS, Park J (2013). Systematic review of basic oral care for the management of oral mucositis in cancer patients. Support Care Cancer.

[7] Hashemi A, Bahrololoumi Z, Khaksar Y, Saffarzadeh N, Neamatzade H, Foroughi E (2015). Mouth-rinses for the prevention of chemotherapy induced oral mucositis in children: a systematic review. Iran J Ped Hematol Oncol.

[8] Nadkerny PV, Ravishankar PL, Pramod V, Agarwal LA, Bhandari S (2015). A comparative evaluation of the efficacy of probiotic and chlorhexidine mouthrinses on clinical inflammatory parameters of gingivitis: a randomized controlled clinical study. J Indian Soc Periodontol.

[9] Marchetti E, Tecco S, Caterini E, Casalena F, Quinzi V, Mattei A, Marzo G (2017). Alcohol-free essential oils containing mouthrinse efficacy on three-day supragingival plaque regrowth: a randomized crossover clinical trial. Trials.

[10] Qiu C, Tang L, Hang Z, Wei K, Wang S, Zhu X, Wang Q (2025). Effect of chlorhexidine vs. saline for oral care on oral microbiota and prognosis of elderly mechanically ventilated patients in the intensive care unit: a single-center, single-blind, randomized controlled trial. BMC Oral Health.

[11] Al-Zawawi AS, Shaheen MY, Divakar DD, Aldulaijan HA, Basudan AM (2022). Postoperative anti-inflammatory efficacy of 2% saline rinses and a herbal- mouthwash after non-surgical periodontal therapy for the management of periodontal inflammation in young adults with chlorhexidine allergy: a randomized controlled trial. Int J Dent Hyg.

[12] Akpata O, Omoregie OF, Owotade F (2013). Alveolar osteitis: patients' compliance to post-extraction instructions following extraction of molar teeth. Niger Med J.

[13] Eid RA (2021). Efficacy of Commiphora myrrh mouthwash on early wound healing after tooth extraction: a randomized controlled trial. Saudi Dent J.

[14] Suparakchinda C, Rawangban W (2023). Effectiveness of Sucralfate comparing to normal saline as an oral rinse in pain reduction and wound healing promotion in oral surgery. Laryngoscope Investig Otolaryngol.

[15] Kozlovsky A, Artzi Z, Hirshberg A, Israeli-Tobias C, Reich L (2007). Effect of local antimicrobial agents on excisional palatal wound healing: a clinical and histomorphometric study in rats. J Clin Periodontol.

[16] Namuangchan Y, Chailertwanich O, Susinsamphan S (2023). Prophylaxis of oral mucositis with iodine solution during concurrent chemoradiation of head and neck cancer: preliminary results of a double-blind, randomized controlled trial. Asian Pac J Cancer Prev.

[17] Ambili R, Ramadas K, Nair LM (2023). Efficacy of a herbal mouthwash for management of periodontitis and radiation-induced mucositis - a consolidated report of two randomized controlled clinical trials. J Ayurveda Integr Med.

[18] Immonen E, Paulamäki L, Piippo H (2025). Oral microbiome diversity and composition before and after chemotherapy treatment in pediatric oncology patients. BMC Oral Health.

[19] Povšič K, Munjaković H, Zayed N, Teughels W, Seme K, Fidler A, Gašperšič R (2026). Electrolyzed saline as an alternative to chlorhexidine: antimicrobial and biofilm volume outcomes in a 4-day non-brushing randomized controlled clinical trial. J Periodontol.

[20] Natto ZS, Bakhrebah MA, Afeef M, Al-Harbi S, Nassar MS, Alhetheel AF, Ashi H (2022). The short-term effect of different chlorhexidine forms versus povidone iodine mouth rinse in minimizing the oral SARS-CoV-2 viral load: an open label randomized controlled clinical trial study. Medicine (Baltimore).

[21] Shanbhag M, Lewis AJ, Srikant N (2026). Evaluation of the effects of various types of mouthwash on enamel and cementum of permanent teeth: an in vitro study. ScientificWorldJournal.

[22] D'Amario M, Foffo G, Grilli F, Capogreco M, Pizzolante T, Rastelli S (2025). Treatments for recurrent aphthous stomatitis: a literature review. Dent J (Basel).

